# Advances in Textile Structural Composites

**DOI:** 10.3390/polym15040808

**Published:** 2023-02-06

**Authors:** Rajesh Kumar Mishra

**Affiliations:** Department of Material Science and Manufacturing Technology, Faculty of Engineering, Czech University of Life Sciences Prague, Kamycka 129, 165 00 Prague, Czech Republic; mishrar@tf.czu.cz

Textile-reinforced structural composites are a major discipline of modern-day research and development. The geometry of the reinforcing textile plays a crucial role in deciding the mechanical performance of textile-reinforced composites. Unlike conventional materials, textile geometrical structures, e.g., yarns or fabrics, can be designed and developed for load bearing in a particular direction [1]. Their properties can be enhanced by modifying the structure and material composition. The challenge in producing textile geometry-based structural composites with superior mechanical properties at a reasonably lower price is cost effective prepreg.

There are many ways of making fabrics from textile fibers. The most common and most complex category comprises fabrics made from interlaced yarns. These are the traditional methods of manufacturing textiles. Great scope lies in choosing fibers with particular properties, arranging fibers in the yarn in several ways, and organizing interlaced yarn in multiple ways within the fabric. This provides the textile designer with greater freedom and variation for controlling and modifying the fabric. The most common form of interlacing is weaving, where two sets of threads cross and interweave with one another. The yarns are held in place due to the inter-yarn friction. Another form of interlacing, where the thread in one set interlocks with the loops of a neighboring thread by looping, is called knitting. The interloping of yarns results in positive binding. Knitted fabrics are widely used in apparel, home furnishings, and technical textiles. Lace, crochet, and different types of net are other forms of interlaced yarn structures. The basic unit of a knitted structure is called a loop. A stitch is formed when one loop is drawn through another loop. Stitches may be formed in horizontal or in a vertical direction. Weft knitting is a method of forming a fabric by means of the intermeshment of horizontal loops in a circular or flat form on a course wise basis. In this method, one or more number of yarns are fed to a group of needles placed in either a lateral or circular fashion. Warp knitting is a method of forming a fabric by the intermeshment of loops made in a vertical way from each warp yarn. In this method, a number of end of yarns are fed simultaneously to individual needles placed in a lateral fashion [2].

Braiding is another way of thread interlacing for fabric formation. Braided fabric is formed by the diagonal interlacing of yarns. Braided structures are mainly used for industrial composite materials. Other forms of fabric manufacture use fibers or filaments laid down, without interlacing, in a web where they are bonded together mechanically or by using an adhesive. The former are needle-punched nonwovens and the later are spun-bonded. The resulting fabric after bonding normally produces a flexible and porous structure. These find use mostly in industrial and disposable applications. All these fabrics are broadly used in three major applications such as apparel, home furnishings, and for industrial uses. The traditional methods of weaving and hand weaving will remain supreme for high-cost fabrics with a rich design content [3]. The woven structures provide a combination of strength with flexibility. The flexibility at small strains is achieved by yarn crimp due to the freedom of yarn movement, whereas at high strains, the threads take the load together, providing a high strength. A woven fabric is produced by interlacing two sets of yarns, the warp, and the weft, which are at right angles to each other in the plane of the cloth. The warp is along the length and the weft is along the width of the fabric. Individual warp and weft yarns are called the ends and picks [4]. The interlacement of the ends and picks with each other produces a coherent and stable structure. The repeating unit of interlacement is called the weave. The structure and properties of a woven fabric are dependent upon the constructional parameters, such as the thread density, yarn fineness, crimp, weave, etc. [5].

The forming of textile reinforcements is an important stage in the manufacturing of textile composite parts. Fiber orientations and part geometry obtained from this stage have a significant impact on the subsequent resin injection and final mechanical properties of the composite part [6]. The numerical simulation of the forming of textile reinforcement is in strong demand as it can greatly reduce the time and cost in the determination of the optimized processing parameters, which is the foundation of the low-cost application of composite materials. The literature presents the state-of-the-art for forming, modeling methods for textile reinforcement and the corresponding experimental characterization methods developed in this field [7]. 

Textile-reinforced composites and fabric-reinforced mortars have emerged as a promising solution for lightweight construction elements and structural retrofit and strengthening. These novel composites consist of two- or three-dimensional textile structures, alkali-resistant glass, or polymer multi-filament yarns, etc. Given the high tensile strength of the textile yarns and their durable nature under normal service conditions, construction elements and strengthening covers reinforced with textile structures feature small thicknesses and offer a higher flexibility in terms of fabrication and application technology as well as the element’s shape [8]. 

The Special Issue provides an overview of the available textile geometrical reinforcements possible for use in composite reinforcement. It dealt with the different types of textile structures for load bearing applications. The uses of industrial multifilament yarns of a pure and hybrid composition in textile structures, e.g., woven, knitted, braided, and multiaxial structures, were summarized [9]. 

In this Special Issue, the microscopic, mesoscopic, and macroscopic models were discussed since this is the most common defect occurring in the manufacturing of textile reinforcement structures. The characterization and analysis of their performance is addressed in detail in this issue.

This Special Issue focused on the application of textile structural composites. The significance and potential of textile composites was described briefly. Given the regularity in the textile preforms, of which textile composites are made, there are plenty of applications reported. Textile-based composites are fast becoming key in many industries, such as automotive, military, aeronautical and aerospace, and construction industries, mainly due to their very attractive specific properties (e.g., strength to weight ratio). Particularly, 3D woven composites (a three-dimensional weaving pattern) have appeared in many new applications requiring high mechanical properties. Evidently, the increasing interest in these materials has generated a high demand for the proper characterization of the methods, accurate FE simulations, suitable nondestructive testing techniques, and adequate visualization techniques. The structured nature of textile-reinforced composites, as described by their geometrical pattern, allows for a new type of analysis, namely one that takes into account their topology [10]. The Special Issue details the various applications of advanced textile structural composites into collective groups.

The Editor is thankful to all the contributors and editorial staff for preparing this Special Issue successfully and effectively. These specialized contributions will lead to new ways of research and development in this area.
